# Emerging concepts in male contraception: a narrative review of novel, hormonal and non-hormonal options

**DOI:** 10.1177/26334941221138323

**Published:** 2023-03-08

**Authors:** C. Austin Service, Dhruv Puri, Tung-Chin Hsieh, Darshan P. Patel

**Affiliations:** Department of Urology, University of California San Diego, San Diego, CA, USA; Department of Urology, University of California San Diego, San Diego, CA, USA; Department of Urology, University of California San Diego, San Diego, CA, USA; Department of Urology, University of California San Diego, 9333 Genesee Avenue, Suite 320, La Jolla, CA 92121, USA

**Keywords:** antispermatogenic agents, condoms, contraception, contraceptive agents, male, spermatogenesis-blocking agents, vasectomy

## Abstract

Access to reliable contraception is a pillar of modern society. The burden of unintended pregnancy has fallen disproportionately on the mother throughout human history; however, recent legal developments surrounding abortion have sparked a renewed interest in male factor contraceptives beyond surgical sterilization and condoms. Modern efforts to develop reversible male birth control date back nearly a century and initially focused on altering the hypothalamic-pituitary-testes axis. These hormonal contraceptives faced multiple barriers, including systemic side effects, challenging dosing regimens, unfavorable routes of delivery, and the public stigma surrounding steroid use. Novel hormonal agents are seeking to overcome these barriers by limiting the side effects and simplifying use. Non-hormonal contraceptives are agents that target various stages of spermatogenesis; such as inhibitors of retinoic acid, Sertoli cell–germ cell interactions, sperm ion channels, and other small molecular targets. The identification of reproductive tract–specific genes associated with male infertility has led to more targeted drug development, made possible by advances in CRISPR and proteolysis targeting chimeras (PROTACs). Despite multiple human trials, no male birth control agents have garnered regulatory approval in the United States or abroad. This narrative review examines current and emerging male contraceptives, including hormonal and non-hormonal agents.

## Introduction

Reliable family planning is an essential element of modern society, with unplanned pregnancies inflicting a significant financial and emotional burden on both the individual and societal levels. For much of human history, the socioeconomic burden of unintended pregnancies has fallen disproportionately on the mother, with its associated financial consequences. The US News and World Report predicts an average cost of $267,000 to raise a child to 18 years of age.^1^ There is also a significant economic burden on the United States taxpayer from unplanned pregnancies, with estimates between $5.5 billion and $21 billion per year.^2–4^

Over the last century, there has been significant progress to ensure safe, reliable, and cost-effective female contraceptives, such as the birth control pill, intrauterine devices (IUDs), emergency contraceptives, diaphragms, vaginal rings, subcutaneous injections, intramuscular (IM) injections, and implants.^5,6^ Despite these efforts, approximately half of all pregnancies are unplanned.^7^ Recent estimates suggest that there are 121 million unintended pregnancies annually worldwide, with 61% of these pregnancies ending in abortion.^8^ Recent data in the United States suggests that 45% of pregnancies are unplanned, and 40% of these pregnancies end in abortion.^9^ With recent rulings in the United States Supreme Court casting doubt on access to legal abortion, there has been a renewed need for reliable family planning methods.^10^

The ability of men to actively participate in family planning with male centered contraceptives has been limited by the number of options, primarily condoms or vasectomy. Estimated numbers from the United Nations Department of Economic and Social Affairs report in 2019 showed that male centered birth control accounted for only 28% of worldwide contraceptive use (male condoms 21%, vasectomy 2%, and withdrawal 5%).^6^ These values are only slightly higher when looking at North America (total 30.9%, male condom 17.6%, vasectomy 6.9%, and withdrawal 6.4%) and Europe (total 39.7%, male condom 29.2%, vasectomy 3%, withdrawal 7.5%).^6^ Over the last half century, there has been an increased interest in developing reliable and safe male birth control options other than the condom or vasectomy. Between 25% and 71% of men report that they would use a male birth control option analogous to the female pill if one was commercially available.^11,12^ Throughout the last several decades, there have been multiple hormonal and non-hormonal birth control options with promising results; however, no option has garnered the US Food and Drug Administration (FDA) approval. In this review, we examine the past and current agents of male birth control research.

## Current forms of male contraception

### Condoms

The origin of male birth control is debated by scholars, but likely dates back to condom use in ancient Egypt and Greece. The first recorded use of a condom is found in the legend of Minos and Pasiphae, written by Antoninus Liberalis in the second century AD. Pasiphae is said to have used a goat’s bladder condom to protect her from evil serpents and scorpions in her unfaithful husband’s semen.^13^ In the 10th century AD, the Persian physician Al-Akhawayni encouraged the use of animal gallbladder condoms. These ‘skin’ condoms were eventually replaced by rubber in 1844 and latex in the 1930s.

Male condom use is associated with a 2–3% unintended pregnancy rate with perfect use and 12% with typical use.^5,14^ There have been many public service campaigns to encourage consistent and proper condom use for the prevention of sexually transmitted infections (STIs) and unintended pregnancies. One national study of 5865 US adolescents and adults aged 14–94 showed that only 21.5% of men had used a condom at least once in the last 10 vaginal intercourse encounters.^15^ Another cross-sectional national survey of adults aged 18–44 showed an overall prevalence of condom use of 24.8% at their last sexual encounter.^16^ In addition, the use of condoms has been shown to be associated with mistrust in relationships or to interfere with intimacy.^17,18^ There are also condoms developed with spermicidal agents, but poor evidence to suggest any greater benefit in preventing unwanted pregnancies.^19^

### Vasectomy

The first documented vasectomy was performed in 1823 by Sir Ashley Cooper on a dog.^20^ Gosselin continued this research with human cadaver studies and further experimenting with vasectomy techniques on dogs in 1847. The first human vasectomy is credited to R. Harrison in London.^20^ Vasectomy was not initially seen for its value as a contraceptive. Multiple surgeons of the day employed vasectomy with the intent of prostatic atrophy. The Austrian physician Eugen Steinach also purported the use of unilateral vasectomy to restore vigor in older gentleman, a concept known as rejuvenescence.^21^ The history of vasectomy took a darker turn when it was identified as a means to forward the eugenics movement. In 1897, A. J. Ochsner performed the first vasectomy in the United States, which he saw as a means of sterilizing criminals and slowing ‘racial degeneration’.^22^ This disturbing work continued in 1902, when Harry C. Sharp sterilized 42 inmates at the Indiana Reformatory to prevent the birth of future criminals.^23^ It was around the time of the Second World War when the vasectomy was recognized as a viable form of consensual male sterilization.^20^

Approximately 500,000 vasectomies are performed annually in the United States, with 5–10% of married men having undergone the procedure.^5,24^ The unintended pregnancy rate in the first year following vasectomy is between 0.02% and 0.1%.^5,25^ This is a relatively quick and reliable method of sterilization with low complication rates of 1–2%.^26^ One of the major barriers for widespread use of vasectomy is the permanence of the procedure, with the associated high cost and uncertainty of vasectomy reversal success. The complication rate of vasectomies is most determined by the procedure volume, with one study reporting that doctors performing more than 50 procedures per year had one third the complication of their counterparts performing less than 10.^27^ The most common complications are hematoma (2%), infection (3–4%), sperm granuloma (40%), and persistent post-vasectomy pain (1–14%).^28^ The reversibility of the procedure is an important consideration for many patients with the success of the procedure being related to the method of vasectomy and the duration of obstruction. For men who underwent a reversal less than 3 years after their initial operation, patency was reported as >95% and a pregnancy rate was reported at 75% with both rates decreasing as duration of obstruction increased.^29^

## Hormonal regulation of spermatogenesis

Normal semen parameters have been established by international studies sponsored by the World Health Organization (WHO), in which 3589 semen samples were analyzed. The selected men were all deemed fertile with a partner’s time to pregnancy of less than 12 months. They defined a normal sperm concentration as greater than 15 million/ml of ejaculate.^30^ With normal semen parameters, 75% of couples will achieve pregnancy within 6 months, and 85% of couples will conceive in 1 year.^31,32^ The perfect male contraceptive would lead to azoospermia, where there are no motile sperm identified on semen analysis. This ideal is often unachievable, and a more realistic real-world goal would be a sperm concentration of less than 1 million/ml. The target of <1 million/ml has been shown to result in a pregnancy rate of less than 1% per year, which is similar to female hormonal contraceptive pills.^33^ This target sperm concentration has been deemed acceptable by the American Society of Andrology.^34^ Beyond efficacy, the other main criteria for an acceptable male contraceptive are reversibility and lack of systemic side effects.^35^

**Figure 1.**
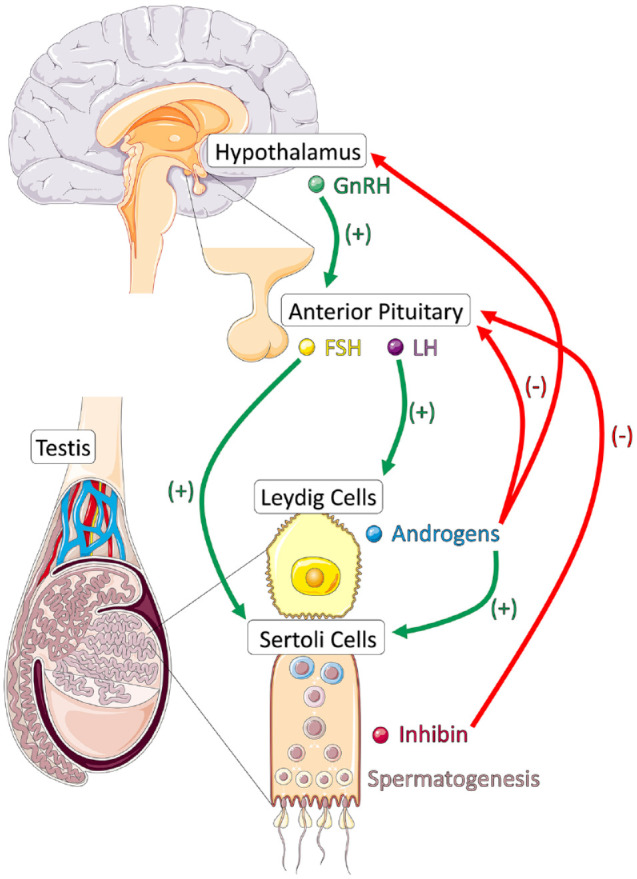
Hypothalamic-pituitary-gonadal axis. Figure modified with text, markings, and annotation after adaptation from Servier Medical Art by Servier, licensed under a Creative Commons Attribution 3.0 Unported License

The first attempts to develop male birth control by lowering sperm levels were targeted at the hormonal axis between the hypothalamus, pituitary gland, and testes. In healthy, eugonadal men, this axis operates with a negative feedback loop to regulate spermatogenesis and steroidogenesis, as shown in Figure 1. Pulsatile release of gonadotropin-releasing hormone (GnRH) from the hypothalamus is transported *via* a portal vascular system to the anterior pituitary.^36^ The gonadotroph cells in the anterior pituitary respond to GnRH by releasing luteinizing hormone (LH) and follicle-stimulating hormone (FSH) into the systemic circulation. FSH acts on the Sertoli cells to stimulate spermatogenesis in the seminiferous tubules. LH acts on Leydig cells to stimulate the production of testosterone, subsequently increasing systemic levels of estradiol and 5-α-dihydrotestosterone (DHT). Testosterone from the Leydig cells and Inhibin from the Sertoli cells work *via* negative feedback to dampen gonadotropin release from the hypothalamus and pituitary. When functioning properly, this process produces several million sperm per day and takes about 68 days for maturation.^37^

## Advent of hormonal options for male birth control

Mankind has known about the effects of androgen deprivation for centuries. Aristotle wrote about the effects of castration and the subsequent inability to reproduce, which is regarded as the first scientific article on infertility.^38^ Arnold Adolph Berthold, the father of modern endocrinology, performed testicular transplantation experiments in 1849 with roosters, where he noted the return of masculine characteristics after the transplantation of testicles into previously castrated roosters.^38,39^ In 1935, testosterone was isolated from bull testes by Ernest Lacquer and simultaneously synthesized in the laboratory by Adolf Butenandt and Leopold Ruzicka.^38^ The idea of targeting the hypothalamic-pituitary-testes axis for male birth control was formulated after witnessing the effects of testosterone supplementation in healthy men. This was done initially with either injections, oral formulations, or topical applications.

### Testosterone enanthate

In 1939, 4 years after the isolation of testosterone, Norris J. Heckel described decreased spermatozoa count in a 67-year-old man with daily subcutaneous injection of 10–25 mg of testosterone enanthate (TE). Sperm counts returned to normal after the cessation of the injections.^40^ The first clinical trials looking at sperm count suppression with testosterone supplementation were conducted in the 1970s at the National Institutes of Health. These studies showed efficacy in causing oligospermia or azoospermia in the majority of participants.^41–43^ The WHO conducted two landmark studies in the 1980s and 1990s. The first was a multicenter, international prospective trial with 271 healthy, fertile men. They injected 200 mg TE IM weekly and noted 65% became azoospermic in 3 consecutive semen samples with a mean time to azoospermia of 120 days. There was one recorded pregnancy during 1486 months of the efficacy phase. Importantly, the time to recovery (sperm concentration of at least 20 million/ml) was 3.7 months after discontinuation and 6.7 months to baseline semen parameters.^44^ The second WHO study included five additional centers, two additional countries, and 399 healthy men. With the same regimen, they noted four pregnancies for 49.5 person-years involving men with oligozoospermia and 0 pregnancies during 230.4 person-years in azoospermic men.^45^ These studies demonstrated efficacy and reversibility of TE injections, but also highlighted several key limitations to this formulation. Azoospermia was only induced in approximately 65% of participants, the frequent injection schedule was not desirable, and there were systemic side effects of unopposed testosterone supplementation; acne, weight gain, altered mood, and changes in hematologic and lipid profiles.^35^

### Testosterone undecanoate

Due to the undesirable injection schedule of testosterone enanthate, there were attempts to study oral formulations of testosterone, namely testosterone undecanoate (TU). The first study with oral TU 80 mg three times daily showed that this dose was insufficient to suppress gonadotropins for sustained azoospermia in six out of seven volunteers.^46^ TU monotherapy was abandoned due to this sperm ‘rebound’.

## Testosterone and progesterone combination therapy

With advancement of male endocrinology, attempts were made to improve sperm suppression, time to suppression, and decrease side effects by using combination regimens of testosterone and progesterone. Multiple of the following regimens also introduce alternate routes of delivery, namely topical gels and implantable pellets.

### Progestin cyproterone acetate

Progestin cyproterone acetate (CPA) is a ‘first-generation’ progestin with antiandrogen effects used for treatment of androgen driven processes such as advanced prostate cancer, acne, precocious puberty, excess body hair growth, female birth control, and puberty blockers in transgender females.^47^ CPA was first tested as a single agent for male contraception in 1980 in 25 men.^48^ Doses of 0, 5, or 10 mg/day were given for 16 weeks, then participants were followed for 24 weeks with semen and serum assays. They noted significant decreases in sperm concentration, motility, morphology, serum testosterone, and gonadotropins. The decrease in androgen levels made this an unlikely candidate for single agent use. Later studies looked at testosterone enanthate in combination with high (25 mg/day) and low (12.5 mg/day) dose CPA for male contraception in the 1990s.^49^ They studied 10 normal men for 16 weeks and noted azoospermia in all men in the high-dose arm and 60% of the low-dose arm. Importantly, there were no changes in serum testosterone levels, and gonadotropin drops were transient. Side effects include gynecomastia, sexual dysfunction, bone density loss, and depression. CPA is no longer used in the United States and has an unlikely future as a component in male birth control.

### Levonorgestrel implants

Levonorgestrel (LNG) is a progestin commonly used in female birth control and emergency contraception. There are multiple routes of delivery for LNG, including oral, skin patch, IUD, and subcutaneous implant.^50^ It was studied as an implant for male contraception in combination with TU injections. This study included 62 Chinese men at three dosage groups. Groups I and II received four LNG subcutaneous rods (75 mg each) followed by either 500 mg or 1000 mg of TU IM every 8 weeks, respectively. Group III was a control, with only 1000 mg TU IM every 8 weeks. They found rates of oligospermia (sperm concentration < 3 million/ml) of 95%, 100%, and 86% in the three groups, respectively. Group II trended toward the highest percentage of azoospermia (90%), but this was not significant. There were no significant side effects or changes in baseline serum testosterone or gonadotropins.

### Etonogestrel implants

Etonogestrel (ENG) has been used since the 1990s as a long-acting, progestin female contraceptive in the form of subcutaneous implants or vaginal rings.^51^ In the early 2000s, ENG was studied as a male contraceptive in a double-blind, multicenter, placebo-controlled study at high and low doses in combination with TU injections.^52^ There were 354 healthy men treated for at least 42 weeks and followed for an additional 24 weeks post treatment. They found that azoospermia was achieved in 89–94% of men by 16 weeks and sustained in 91% of men on treatment. Recovery to sperm concentrations > 20 million/ml was 15 weeks. Side effects of this treatment included weight gain, mood changes, acne, sweating, and loss of libido.

### Desogestrel

Desogestrel is an oral progestin used in combination with an estrogen agent or individually in female birth control and to treat menopausal symptoms.^53,54^ A multicenter, prospective, randomized trial was conducted with 38 Caucasian and 36 Chinese men to look at the use of low (150 µg daily) and high (300 µg daily) dose oral desogestrel and testosterone pellets (400 mg weeks 1 and 12).^55^ The high dose group achieved azoospermia at a significantly higher rate than the low dose group (100% *versus* 71%, *p* < 0.05). The testosterone concentrations remained normal, but side effects included lower HDL in caucasian participants and more weight gain in both groups.

### Norethisterone acetate PO or norethisterone enanthate IM

Norethisterone is a progestin with both oral (Norethisterone Acetate, NETA) and IM injection (Norethisterone Enanthate, NETE) formulations. A phase II clinical trial was conducted looking at NETE or NETA in combination with TU injections.^56^ Groups I and II received low-dose (200 mg) or high-dose (400 mg) NETE every 6 weeks with 1000 mg TU every 6 weeks, respectively. Group III received 10 mg daily NETA with 1000 mg TU every 6 weeks. These regimens produced sperm concentrations < 1 million/ml in all participants with azoospermia in 13/14 (group I), 11/12 (group II), and 12/14 (group III). The side effects included increase in body weight, erythrocytes, hemoglobin, and hematocrit and decrease in HDL and alkaline phosphatase. They also noted an increase in liver enzymes in the oral NETA group.

### Medroxyprogesterone acetate

Medroxyprogesterone acetate (MPA) is an oral or injectable progestin used commonly in depot formulation for female birth control. It also treats menopausal symptoms, endometriosis, abnormal uterine bleeding, certain cancers, precocious puberty, and male paraphilia.^47,57^ The depot formulation (300 mg IM every 3 months) was studied in tandem with testosterone implants (every 3 or 4 months) in 55 healthy men as a form of long-acting male birth control.^58^ Once the men reached azoospermia, they entered a 12 month efficacy period. They reported no pregnancies in 426 person-months. There were two men (3.6%) who did not reach azoospermia. Once the efficacy period was terminated, there was complete recovery to sperm concentration > 20 million/ml in 5 months.

## Novel hormonal agents

Prior testosterone and progesterone treatment options have been inconvenient for study participants due to the multiple routes of delivery and dosing regimens. Current hormonal options are not ideal for patients due to several factors including adverse side effect profiles of testosterone excess, failure rates of current hormonal agents, and rates of recovery of spermatogenesis.^59^ Novel hormonal agents have aimed to simplify the route of delivery and dosing regimen to make them more convenient for patients.

### Nestorone and testosterone gels

Segesterone acetate (SGA) is a progestin that has no androgenic, estrogenic, or glucocorticoid activity. Nestorone (NES), the brand name equivalent of SGA, has been formulated as a transdermal gel, making it a possible companion for combined testosterone and progestin gel birth control. One of the first male birth control studies to employ daily NES gel analyzed the effect on serum gonadotropin levels with and without combined testosterone gel in 2009.^60^ This was a randomized, unblinded, dual-center study with 140 healthy men. There were seven groups of 20 men who received between 2 and 8 mg of daily NES gel with or without 10 mg testosterone gel. They found the highest levels of gonadotropin suppression (<0.5 IU/L) in the combined groups, suggesting a need for further research into effects on sperm concentration with the combined NES plus testosterone regimen.

The first study to look at the effects of NES gel plus testosterone gel on sperm concentrations was in 2012.^61^ This was a randomized, double-blind, dual-center study with 99 healthy men. There were three treatment groups, each with 10 mg testosterone gel plus 0, 8, or 12 mg NES gel. These were administered as two gels. The primary endpoint was azoospermia at 20–24 weeks. They found similar rates of azoospermia in the testosterone plus 8 mg NES group and testosterone plus 12 mg NES group (89% and 88%, respectively). These were significantly higher than the placebo group (23%, *p* < 0.0001 and *p* = 0.0002). The median serum free testosterone and total testosterone were also maintained in the normal range. Only 57% adhered to the protocol, prompting the group to look at patient satisfaction. Serious side effects were minimal, five men discontinued the trial due to irritability, nightmares, decreased libido, increased appetite, mood swings, and asthma exacerbation. The remaining participant discontinuations were due to inconvenience of the dosing regimen and frequency of study related visits. In 2014, the group reported a 56% satisfaction with the regimen, and only 33% said they would use the two gels as their primary form of contraception.^62^

In order to improve compliance, a single gel formulation with combined NES and testosterone was developed (NES-T).^63^ This daily, single gel birth control was studied in 44 healthy men in a double-blind, controlled trial.^63^ They found that 84% of the NES-T group had suppressed serum gonadotropins by day 28 compared to 16.7% in the T-only gel group (*p* < 0.001). Importantly, 80% of the men were satisfied with the regimen and over 50% said they would use the single gel formulation as their primary form of contraception if available on the market.

### Dimethandrolone undecanoate

Dimethandrolone undecanoate (DMAU) is a synthetic precursor drug of dimethandrolone (DMA), which acts on both androgen and progesterone receptors in the body. The dual androgen and progesterone activity have made it an appealing target for single agent male birth control. There are oral and IM formulations of this drug. A randomized, double-blind, phase I trial in 2014 looked at safety for doses of DMAU between 25 and 800 mg *versus* placebo.^64^ At doses above 200 mg of oral DMAU, serum gonadotropins and sex hormones were significantly depressed when taken with food. There were no serious adverse events, changes in vital signs, or laboratory changes up to concentrations of 800 mg. In 2019, a double-blind, randomized, placebo-controlled study with 100 healthy men looked at serum testosterone and gonadotropin levels at various doses of oral DMAU for 28 days.^65^ They found that doses of 200 mg per day were sufficient to markedly reduce serum testosterone, LH, and FSH. Acceptability surveys showed that 87% of men receiving the active medication were satisfied with this birth control method, 91% reported no difficulty taking the pills with a high-fat meal, and more than half would use it as their primary form of contraception.^66^

### 7α-Methyl-19-nortestosterone

7α-Methyl-19-nortestosterone (MENT) is a synthetic androgen with activity at both androgen and progesterone receptors.^67^ It is more potent than testosterone and has more resistance to 5-α-reductase.^68^ MENT acetate was developed as a long-term birth control using ethylene vinyl acetate implants. This drug was studied in the early 2000s in 35 men who received between one and four subdermal implants (each implant released approximately 400 µg per day). These were maintained for 6–12 months. They found an increase in MENT serum levels with suppressed testosterone, LH, and FSH levels. Azoospermia occurred in 67% (8/12) of the men in the four-implant group. In 2007, MENT acetate implants were studied in combination with ENG implants *versus* testosterone pellets with ENG implants.^69^ They achieved azoospermia in 80% of men in the MENT group within 12 weeks. Unfortunately, suppression was not maintained due to decrease in MENT release from the implants over time. Side effects included loss of libido in 60% of men. They also noted decreased serum prostate-specific antigen (PSA) with no change in bone mass, which is due to the resistance to 5-α-reduction and lower activity in the prostate.

### 11β-Methyl-19-nortestosterone-17β-dodecylcarbonate

11β-Methyl-19-nortestosterone-17β-dodecylcarbonate (11β-MNTDC) is a novel androgenic steroid with progestogenic activity, identified for its specificity in suppressing spermatogenesis with a lack of systemic side effects. It is a prodrug of 11β-methyl-19-nortestosterone (11β-MNT), the biologically active compound. 11β-MNTDC does not undergo aromatization and therefore has fewer expected estrogenic effects. It is also more resistant to 5-α-reduction and therefore has fewer expected androgenic side effects than testosterone. This drug was studied in a randomized, double-blinded, placebo-controlled, phase I clinical trial in 2019.^70^ Single oral doses (0, 100, 200, 400, and 800 mg) were given with or without food. They found that ingestion with food significantly increased serum 11β-MNTDC and 11β-MNT levels. They found that doses up to 800 mg were safe and doses between 200 and 800 mg were sufficient to significantly suppress serum testosterone levels. Future studies are warranted to examine gonadotropin suppression and its effects on spermatogenesis.

### GnRH antagonists

GnRH antagonists act by suppressing LH and testosterone through competitive inhibition of pituitary GnRH receptors. In a randomized clinical trial in 2004, different GnRH antagonist preparations were found to cause azoospermia in 39 of 47 subjects, and more recently, acycline has been tested in contraceptive trails, although its primary use is in the treatment of prostate cancer.^71^ Despite promising results, GnRH antagonists require daily or weekly injections, and incur high costs, leading to many drug developers to not pursue them further (Table 1).

**Table 1.** Summary of novel hormonal agents.

Drug category and name	Citation	Number of subjects	Drug dose, route of administration, and timing	Outcome measured
Nestorone (NES) and testosterone gels	Inhibitory effect on serum gonadotropin levels			
	Mahabadi *et al.*^60^	140	2 and 8 mg of daily NES gel with or without 10 mg testosterone gel	Subjects showed highest levels of gonadotropin suppression (<0.5 IU/L) when receiving 8 mg NES gel with testosterone.
	Ilani *et al.*^61^	99	10 mg testosterone gel plus 0, 8, or 12 mg NES gel	Subjects showed similar rates of azoospermia in the testosterone plus 8 mg NES group and testosterone plus 12 mg NES group (89% and 88%, respectively)
	Anawalt *et al.*^63^	44	Daily, single gel with combined NES and testosterone	84% of the NES-T group had suppressed serum gonadotropins by day 28 compared to 16.7% in the T only gel group (*p* < 0.001).
Dimethandrolone undecanoate (DMAU)	A synthetic 1-norandrogen that is a prodrug of dimethandrolone (DMA), an agonist of both androgen and progesterone receptors.			
	Surampudi *et al.*^64^	12	DMAU (25 and 800 mg)	At doses above 200 mg oral DMAU, serum gonadotropins and sex hormones were significantly depressed.
	Thirumalai *et al.*^65^	100	Daily oral administration of DMAU (at different concentrations) for 28 days	Doses 200 mg markedly suppressed serum T, LH, and FSH in subjects.
7-Alpha-methyl-19-nortestosterone (MENT)	A synthetic androgen with activity at the androgen and progesterone receptors causes testosterone suppression			
	Walton *et al.*^69^	35	Received between 1 and 4 subdermal implants (each implant released approximately 400 µg per day)	The drug led to azoospermia in 80% of men within 12 weeks
11β-methyl-19-nortestosterone-17b-dodecylcarbonate (11β-MNTDC)	A novel androgenic steroid with progestogenic activity, identified for its specificity at suppressing spermatogenesis with lack of systemic side effects.			
	Wu *et al.*^70^	43	Single oral doses (0, 100, 200, 400, and 800 mg)	Doses between 200 and 800 mg were sufficient to significantly suppress serum testosterone levels in subjects.

DMA, dimethandrolone; DMAU, dimethandrolone undecanoate; FSH, follicle-stimulating hormone; LH, luteinizing hormone; NES, Nestorone; NES-T, NES and testosterone.

## Novel non-hormonal agents

Since the 1950s, researchers have realized that spermatogenesis can be suppressed without directly acting on the hypothalamus-pituitary-testes axis. Multiple non-hormonal agents have been identified that either irreversibly or reversibly disrupt sperm maturation. These agents may be good candidates for male contraception when compared to hormonal agents, avoiding the side effects that occur when altering the hormonal pathways, the stigma of hormone supplementation (anabolic steroid use in sports), and the difficulties of the dosing regimen or route of administration of hormonal agents.

These non-hormonal agents can theoretically act at any stage of spermatogenesis. Common targets include the testicular retinoic acid receptor, Sertoli cell–germ cell interactions, testicular epigenetics, and an assortment of small molecule inhibitors.

## Retinoic acid inhibitors

### WIN 18,446

Retinoic acid plays an essential role in spermatogenesis, assisting with development of the blood–testis barrier (BTB), spermatogonial differentiation, and spermiation.^72^ Male mice with knock-outs of the retinoic acid receptor α (RARα) are infertile.^73^ In the late 1950s, researchers studying antiparasitics noted that rats receiving retinoic acid inhibitors developed the unintended consequence of infertility. This realization led to human studies of retinoic acid inhibitors as the first potential non-hormonal male contraceptives.^74^ During the initial human studies at the Oregon State Penitentiary, approximately 60 inmates remained azoospermic for 1 year while receiving the drug designated WIN 18,446. Unfortunately, when one of the inmates obtained access to contraband whiskey, they became incredibly ill with what was later identified as a disulfiram reaction. This is due to the mechanism of the drug, targeting aldehyde dehydrogenase 1A2 in the testes to block the production of retinoic acid. Off-target action on aldehyde dehydrogenase 2 in the liver causes accumulation of serum acetaldehyde, with the associated systemic symptoms. The use of WIN 18,446 was ultimately abandoned, but recent attempts have been made to develop retinoic acid receptor antagonists more specific to the testes.

### BMS-189453

Bristol-Myers-Squibb (BMS) has developed a series of retinoic acid receptor antagonists with varying degrees of specificity to the testes. BMS-189453 is a panretinoic acid receptor antagonist developed in the 1990s with action on the α, β, and γ receptors.^75,76^ In rat models, administration of doses between 5 and 240 mg/kg daily of BMS-189453 resulted in marked testicular degeneration and atrophy.^75^ Doses higher than 240 mg/kg resulted in severe toxicity and death. Other studies looked at lower doses of BMS-189453. Chung *et al.*^77^ used 5 mg/kg for 2 weeks or 2.5 mg/kg for 4 weeks and found that all mice were sterile at 4 weeks with return of fertility by 20 weeks. Even doses as low as 1 mg/kg for 4 to 16 weeks resulted in 100% sterility with a return of fertility after termination of the drug.^78^

Other studies have looked at more α-selective antagonists such as BMS-189532 and BMS-189614.^79,80^ Despite promising *in vitro* studies, when given to mice at 2 and 10 mg/kg orally for 7 days, these medications showed lower potency than non-selective antagonists.^76,80^ Normal spermatid formation and sperm release were observed when given orally, but expected defects in spermatogenesis were seen with intravenous formulations. This suggests that factors, such as hepatic metabolism, higher plasma protein binding, or decreased testicular permeability are decreasing the bioavailability of the α-selective agents.

### YCT529

YCT529 is an α-selective retinoic acid antagonist recently presented at the American Chemical Society national meeting 2022.^81^ When given to mice orally for 4 weeks, it reduced sperm counts and prevented 99% of pregnancy. There were no systemic side effects seen with less specific retinoic acid receptor antagonists. Importantly, mice could reproduce for 4–6 weeks after stopping the medication. Reportedly, human clinical trials will begin this year.

## Targeting sertoli cell–germ cell interactions

### Indenopyridine derivatives

Indenopyridine derivatives, such as CDB-4022 and RTI-4587-073(l), have been shown to inhibit mature sperm production in rat, primate, and stallion models.^82–84^ These molecules work primarily by disrupting the attachment of immature spermatids from the seminiferous tubules, causing sloughing of immature, non-motile germ cells into the semen. Hild *et al.*^82^ studied the effects of a single dose of CDB-4022 on rat testicle ultrastructure. They noted degenerative changes in both Sertoli cells and spermatids. The Sertoli cells had an increase in the number of vacuoles, cellular debris, swollen mitochondria, and swollen endoplasmic reticulum. The spermatids had diffuse chromatin and broken nuclear envelopes. Primate studies with the same molecule showed sperm concentrations lower than 1 million/ml by 17 days and remained suppressed for 6 weeks.^83^ More interestingly, they found sperm motility had dropped to 0% with immature spermatids present. Serum Inhibin B was elevated, but testosterone, LH, FSH, and estradiol were within normal ranges. All parameters of sperm health and serum markers returned to normal by 17 weeks.

The indenopyridine derivative RTI-4587-073(l) was studied in miniature stallions.^84^ They noted severe oligoasthenozoospermia with high numbers of immature germ cells in the ejaculate. They also noted increased FSH concentrations in the treated stallions, reflecting the drugs activity on Sertoli cells. The semen parameters and serum gonadotropin concentrations were fully reversible in about 70 days.

### Lonidamine derivatives

Analogues of Lonidamine, a chemotherapy drug, have been analyzed as potential reversible male birth control agents. Adjudin, one such Lonidamine analogue, is derived from 1H-indazole-3-carboxylic acid. It works by disrupting the Sertoli-germ cell junction by targeting the apical ectoplasmic specialization proteins.^85,86^ This leads to the exfoliation of immature spermatids. Adjudin was studied in a rat model with two doses of 50 mg/kg weekly, which caused infertility in 100% of subjects.^87^ Unfortunately, the target proteins of this drug were not specific to the gonads, and side effects included liver inflammation. This group attempted to increase testicular specificity by conjugating Adjudin with the carrier molecule FSH-β. They showed that this approach successfully increased gonadal selectivity and decreased the effective dose from 50 to 0.50 µg/kg in the rat model.^87^

The BTB plays an important role in regulating all non-hormonal birth control agents. Recent rat studies have shown that overexpressing F5-peptide, an endogenous BTB modulator, can enhance the bioavailability of Adjudin in the testis and lower the needed dose to induce reversible infertility.^88^ By lowering the effective dose of Adjudin, this may lower the systemic side effects to an acceptable level. To date, there are no published *in vivo* human studies with Adjudin.

Gamendazole is a Lonidamine analogue currently being studied for its antispermatogenic properties. This molecule works by inhibiting the heat shock protein HSP90AB1 and the eukaryotic translation elongation factor EEF1A1, leading to increased Interleukin 1α production by Sertoli cells.^89^ Interleukin 1α disrupts the Sertoli cell-germ cell junction, leading to premature exfoliation of immature spermatids, similar to Adjudin. There is also a noted decrease in Inhibin B and associated increase in FSH. Initial rat studies demonstrated 100% infertility 3 weeks after a single dose of 6 mg/kg.^90^ Unfortunately, fertility only returned to 57% of the initial cohort. Doses of 3 mg/kg resulted in 66% infertility and 100% return of fertility. Further studies into dosing and reversibility need to be completed prior to human studies.

## Sperm ion channel blockers

CatSper is a calcium ion channel specific to sperm flagella and is essential for sperm motility.^91,92^ It has roles in flagella hyperactivity, chemotaxis toward the female ovum, capacitation, and the acrosome reaction.^92^ There are four CatSper channels that have all been shown to be critical to male fertility in mouse knock-out models.^91,93,94^ Below are several promising sperm CatSper blocker targets. Other sperm-specific ion channels, such as the potassium channels KSper and SLO3, are also being investigated as potential targets.

### RU1968

RU1968 is a synthesized inhibitor of CatSper and SLO3.^95^ Cross-species studies, including sperm from humans, mice, and sea urchins, showed inhibition of CatSper with a 15-fold higher potency than SLO3.^95^ No toxic side effects were seen in human sperm. The mechanism of action is still being elucidated, but RU1968 was shown to inhibit the pro-motility response caused by progesterone in the female reproductive tract.^95,96^

### HC-056456

HC-056456 is a CatSper blocker shown to reversibly and selectively decrease ion transit through the CatSper channel of patch-clamped sperm.^91^ When treated with HC-056456, human sperm loses flagellar hyperactivity.^91^ *In vivo* studies in a mouse model found decreased fertilization in HC-056456 treated sperm when inseminated into the uterus.^97^ This was the first *in vivo* study to look at CatSper blockers as a potential male contraceptive in a mammalian model.

### SLO3 channel inhibitors

SLO3 is a sperm-specific potassium channel that regulates calcium entry through the CatSper channel.^98^ SLO3 works by hyperpolarizing the cell during capacitation to allow for calcium entry *via* CatSper. It may also work indirectly by altering the pH of sperm. Knock-out mice lacking SLO3 are infertile.^98^ Research is being done to look at potential SLO3 inhibitors, such as quinine and quinidine, but to date there are no human trials.^99^

### Pristimerin and lupeol

Pristimerin and lupeol are plant triterpenoids that are thought to inhibit sperm hyperactivation *via* the CatSper channel. Pristimerin is an isolate from *Tripterygium Wilfordii*, and Lupeol is isolated from dandelion root, aloe vera, and mangos. They are thought to work by binding CatSper and blocking activation by progesterone and pregnenolone.^100^ Further research has cast doubt on the efficacy of these medications at inhibiting sperm hyperactivation, and further research is still needed to determine their potential as male contraceptives.^96^

## Small molecule inhibitors

An emerging area of interest is small molecule inhibitors of the spermatogenesis pathway. These molecules act by blocking the action of target proteins, typically enzymes. Below are examples of small molecule inhibitors that act in the male reproductive tract.

### JQ1

JQ1 is a small molecule inhibitor of a testicular bromodomain protein named BRDT.^101^ Bromodomain proteins are involved in epigenetics by assisting with histone acetylation, chromatin remodeling, and recruiting transcription factors.^102^ BRDT knock-out mice are healthy but sterile.^103^ Mouse models suggest that JQ1 leads to infertility by reducing the number of spermatozoa, sperm motility, and seminiferous tubule volume without altering serum hormone levels.^101^ Male mice pretreated with JQ1 for 6 weeks were unable to procreate when caged with female mice continuously.^104^ These effects were reversible, but JQ1 had off-target effects on non-testicular bromodomain proteins.^105^ Future research will need to advance testicular specificity.

### EP055

EP055 is a small molecule inhibitor of Eppin, an epididymal protease inhibitor.^106^ Eppin is secreted by Sertoli cells and binds to the sperm surface in the testes. It is bound by semenogelin from the seminal vesicle during ejaculation to modulate sperm motility. Eppin then regulates PSA enzyme activity to hydrolyze semenogelin and induce progressive motility.^107^ In male nonhuman primates immunized with Eppin, 78% developed high titers to Eppin and were infertile. After cessation of immunocontraception, 71% recovered fertility.^108^ Further primate studies showed that infusion with low dose EP055 (75–80 mg/kg) followed by high dose (125–130 mg/kg) led to no normal sperm motility 30 h after initial infusion and full recovery of sperm motility by 18 days.^106^

### Calcineurin inhibitors

Cyclosporine A and FK506 (tacrolimus) are calcineurin inhibitors used as immunosuppressant drugs.^109^ Their adverse effect on male fertility has been observed and led to research into their efficacy as reversible non-hormonal male contraceptives. The sperm-specific calcineurin subunits PPP3CC and PPP3R2 are potential targets for inhibition, as mice with knock-out in these genes are infertile with reduced sperm motility.^109,110^ The mechanism is believed to be the inflexibility of the sperm midpiece. When healthy mice are treated with Cyclosporine A or FK506, they develop defects of sperm motility and morphology within 4–5 days. These effects are reversible within 7 days of stopping the medication.^109^ Testis-specific calcineurin inhibitors may be a viable form of reversible male contraception. Other molecules essential to the sperm calcineurin pathway may also be targetable. Inhibition of SPATA33, which localizes calcineurin to the mitochondria, has also led to infertile knock-out mice with reduced sperm motility.^111^

## Inhibitors of vasal peristalsis

Inhibition of the peristalsis of sperm through the male reproductive tract is an additional mechanism that has been explored for possible male contraceptives. Early studies explored phenoxybenzamine, an alpha-1-adrengergic antagonist, for its contraceptive characteristics through the selective blockade of the longitudinal muscles of the vas deferens and inhibition of peristalsis.^112^ It was previously shown to cause reversible infertility of rats and block ejaculation in a small cohort of adult males.^113,114^ Studies have explored other alpha-1-adrenergic antagonists as well, namely prazosin and tamsulosin. Despite prazosin’s debated efficacy as a contraceptive agent, tamsulosin has previously been shown to decrease sperm concentration; albeit with side effects such as dizziness and orthostatic hypotension.^115–119^ To date, there have been no large-scale clinical trials evaluating alpha-1-adrenergic antagonists as potential male contraceptives. Most recently, P2X1-purinoreceptor antagonists have been postulated as another option. P2X1-purinoreceptors are adenosine triphosphate (ATP) ligand–gated cation channels expressed on the cell membranes of smooth muscle cells along the vas deferens, and their absence leads to impaired peristalsis and reduced sperm concentration in the semen.^120,121^ Eise *et al.*^122^ showed that a selective blockage of P2X1-purinoreceptors, through an oral agent in rats, led to a reduced number of pregnancies during mating. It has yet to be further evaluated in large-scale clinical trials as well.

## Other inhibitors of spermatogenesis

### Triptonide

Triptonide is a traditional Chinese herb produced from the vine of *T. Wilfordii*, also known as thunder god vine. Observational studies looking at *Tripterygium* use for rheumatoid arthritis in humans showed lower sperm counts.^123^ Triptonide use in mice and primates caused morphological defects and sperm with no forward motility.^124^ Infertility was seen by 3–4 weeks in male mice and 5–6 weeks in cynomolgus monkeys after initiation. Both species regained fertility in 4–6 weeks after stopping the medication. There were no serious systemic effects or changes in serum markers in these studies. It appears to disrupt spermatogenesis by targeting the interaction between junction plakoglobin and SPEM1 (spermatid maturation 1) receptor on the sperm.^124^ Mice with SPEM1 gene knock-out have deformed sperm with a characteristic kinked head wrapped around by the mid-section of the tail.^125^ Naturally occurring compounds causing infertility may continue to offer insight into future male contraceptive pathways.

### Gendarussa leaves

*Justicia gendarussa* Burm f. (Acanthaceae), colloquially known as the gendarussa leaf, is a medicinal plant local to tropical areas in southeast Asia and the Pacific islands and has been used as a method of male contraception by many from the region.^126^ It has also been explored for its potential use in a variety of conditions, including, but not limited to, hypertension, AIDS, arthritis, and liver disease.^127–130^ Specifically, the active ingredient, flavonoid gendarusin A, has shown to reversibly inhibit the activity of spermatozoa hyaluronidase, an enzyme crucial to sperm penetration of the cumulus oophorus during the acrosome reaction.^131–133^ The chemical make-up of the leaf has been explored previously, but its potential use as a male contraceptive has yet to be demonstrated in dedicated preclinic contexts and clinical trials.^134–136^

### Histone demethylases

Histone demethylation plays a crucial role in germ cell regulation in both mice and humans.^137^ KDM5B, a histone demethylase, and several other small molecules that target histone demethylases (CPI-455, PBIT, and KDM5-C70) have been identified as potential targets for male contraception (Table 2).

**Table 2.** Summary of novel non-hormonal agents.

Category and drugs	Citation	Trial model(s)	Number of subjects	Drug dose, route of administration, and timing	Outcome measured
Retinoic acid inhibitors	Retinoic acid plays an essential role in spermatogenesis, assisting with development of the blood-testis barrier, spermatogonial differentiation, and spermiation				
WIN 18,446	Heller *et al.*^74^	Human	60	Oral dose	Azoospermia in subjects
BMS-189453	Schulze *et al.*^75^	Rat	N/A	Single oral doses between 5 mg/kg and 240 mg/kg	Marked testicular degeneration and atrophy seen in rats.
	Chung *et al.*^77^	Mouse	N/A	5 mg/kg for 2 weeks or 2.5 mg/kg for 4 weeks	All mice were sterile at 4 weeks with return of fertility by 20 weeks.
YCT529	ACS^81^	Mouse	N/A	Taken orally for 4 weeks	Drugs led to reduced sperm counts and prevented 99% of pregnancies. Return to normal fertility was seen 4–6 weeks after stopping the medication.
Indenopyridine derivatives (CDB-4022 and RTI-4587-073)	Hild *et al.*^82^	Rat	N/A	Single oral dose of 12.5 mg/kg	Spermatids had an increase in the number of vacuoles, cellular debris, swollen mitochondria, and swollen endoplasmic reticulum.
	Hild *et al.*^83^	Primate	N/A	12.5 mg/kg per day *via* NGT	Subjects showed sperm concentrations lower than 1 million/ml by 17 days and remained suppressed for 6 weeks with sperm motility dropping to 0% with immature spermatids present.
	Pozor *et al.*^84^	Stallions	N/A	Single dose of 12.5 mg/kg	Subjects showed severe oligoasthenozoospermia, shedding large numbers of immature germ cells in semen, and increased FSH concentrations. These effects were fully reversible within ∼71 days.
Lonidamine derivatives	Adjudin works by disrupting the Sertoli-germ cell junction by targeting the apical ectoplasmic specialization proteins, leading to exfoliation of immature spermatids. Gamendazole works by inhibiting heat shock protein HSP90AB1 and eukaryotic translation elongation factor EEF1A1, leading to increased Interleukin 1alpha production by Sertoli cells				
Adjudin	Mruk *et al.*^87^	Rat	N/A	Two doses of 50 mg/kg weekly of adjudin	100% infertility after 5 weeks of treatment without changes in serum hormones or gonadotropins.
Gamendazole	Tash *et al.*^90^	Rat	N/A	Single dose of 6 mg/kg of gamendazole	100% infertility seen 3 weeks post treatment.
Sperm ion channel blockers	CatSper is a calcium ion channel specific to sperm flagella and is essential for sperm motility. There are four CatSper channels that have all been shown to be critical to male fertility.				
RU1968	Rennhack *et al.*^95^	Human, mice, and sea urchin sperm	N/A	Solution of RU1968	RU1968 inhibited CatSper in sperm from invertebrates and mammals. RU1968 mimicked CatSper dysfunction and suppressed motility responses evoked by progesterone, an oviductal steroid known to activate CatSper. Finally, RU1968 abolished CatSper-mediated chemotactic navigation in sea urchin sperm.
HC-056456	Carlson *et al.*^91^	Human sperm	N/A	Solution of HC-056456	When applied to hyperactivated sperm, HC-056456 causes a rapid, reversible loss of flagellar movement.
	Curci *et al.*^97^	Mice	N/A	1–20 μM of HC-056456	Exposure of sperm to HC severely reduced fertilization, and insemination of HC-treated sperm into the uterus significantly or completely reduced the percentage of oviductal fertilized eggs.
Pristimerin and lupeol	Mannowetz *et al.*^100^	Human sperm	N/A	1 μM of pristimerin and lupeol	Both compounds significantly reduced CatSper activation by either steroid. Furthermore, they also considerably diminished hyperactivation of capacitated spermatozoa.
JQ1	JQ 1 is a small molecule inhibitor of a testicular bromodomain protein named BRDT, involved in epigenetics. Mouse models suggest that JQ1 leads to infertility by reducing the number of spermatozoa, sperm motility, and seminiferous tubule volume without altering serum hormone levels				
	Matzuk *et al.*^101^	Mice	N/A	50 or 75 mg/kg of JQ1	Treatment of mice with JQ1 reduced seminiferous tubule area, testis size, and spermatozoa number and motility without affecting hormone levels. The inhibitory effects of JQ1 cause a complete and reversible contraceptive effect. Male mice pretreated with JQ1 for 6 weeks were unable to procreate when caged with female mice continuously.
EP055	EP055 is a small molecule inhibitor of Eppin, an epididymal protease inhibitor secreted by Sertoli cells that binds to sperm in the testes. It is bound by semenogelin from the seminal vesicle during ejaculation to promote sperm motility.				
	O’Rand *et al.*^108^	Nonhuman primates (*Macaca radiata*)	9	Human recombinant Eppin Immunization	78% of primates developed high titers to Eppin and were infertile. After cessation of immunocontraception, 71% recovered fertility.
	O’Rand *et al.*^106^	Rhesus monkeys (*Macaca mulatta*)	4	IV infusion of a single dose of compound EP055 (63.25 mg/kg)	Sperm motility fell to approximately 20% of pretreatment levels within 6 h post-infusion; no normal motility was observed at 30 h post-infusion. Recovery of sperm motility was obvious by 78 h post-infusion; with full recovery in all animals by 18 days post-infusion.
Calcineurin inhibitors	Cyclosporine A and FK506 (tacrolimus) are calcineurin inhibitors used as immunosuppressant drugs. Their adverse effect on male fertility has been observed and led to research into their efficacy as reversible non-hormonal male contraceptives				
Cyclosporine A and FK506 (tacrolimus)	Miyata *et al.*^109^	Mice	N/A	Treated with CsA or FK506 for 2 weeks	Treatment of mice with cyclosporine A or FK506 alters sperm motility and causes morphological defects. Male mouse fertility recovered a week after we discontinued treatment.
Inhibitors of vasal peristalsis	Inhibit vas deferens smooth muscle contraction *via* blockade of alpha-1 or P2X1 receptors				
Phenoxybenzamine	Homonnai *et al.*^114^	Human	N/A	Doses of 20 mg/day PBZ	After treatment for 2–3 days, ejaculation was blocked and was fully reversible after cessation.
Tamsulosin and alfuzosin	Hellstrom and Sikka^118^	Human	48	Received 5 days of tamsulosin 0.8 mg daily, alfuzosin 10 mg daily, or placebo in three-way crossover study	Percentage of motile sperm decreased 13.8% from baseline with tamsulosin, 0.4% with alfuzosin, and 2.3% with placebo. The total sperm count dropped with tamsulosin by 54.6 ± 24 million.
P2X1-receptor antagonists	Eise *et al.*^122^	Rats	N/A	Daily oral dose of 50 mg	Reduced male fertility by 53% compared to control rats. Bladder and testes weighed less in treated animals. Mating behaviors were unchanged.
Triptonide	Triptonide appears to disrupt spermatogenesis by targeting the interaction between junction plakoglobin and SPEM1 (spermatid maturation 1) receptor on the sperm.				
	Chang *et al.*^124^	Mice and cynomolgus monkeys	12 monkeys and 20 mice	Single daily oral doses of triptonide at four doses (0.05, 0.1, 0.2, 0.8, and 5 mg/kg BW).	Triptonide induces deformed sperm with minimal or no forward motility (close to 100% penetrance) and consequently male infertility in 3–4 and 5–6 weeks in mice and cynomolgus monkeys, respectively. Male fertility is regained in 4–6 weeks after cessation of triptonide intake in both species.
Gendarussa leaves	Flavonoid gendarusin A reversibly inhibits the activity of spermatozoa hyaluronidase, an enzyme crucial to sperm penetration of the cumulus oophorus during the acrosome reaction. Has not been studied in thorough mammalian studies.				

ACS, American Chemical Society; BMS, Bristol-Myers-Squibb; FSH, follicle-stimulating hormone; SPEM1, spermatid maturation 1.

## Identifying future non-hormonal targets

There are more than 1500 human genes associated with male infertility, of which 200 are believed to be reproductive tract specific.^138^ These genes and proteins offer a host of enticing targets for future non-hormonal male contraceptives. Sinha *et al.*^139^ have recently reported on the development of a searchable database to help researchers identify high-quality contraceptive targets (Contraceptive and Infertility Target DataBase, https://www.citdbase.org). Current advances in PROTAC (proteolysis targeting chimera) and CRISPR (clustered regularly interspaced short palindromic repeats) technologies have unlocked the ability to eliminate specific proteins and knockout specific genes, respectively. PROTACs eliminate proteins *via* the ubiquitin-proteasome pathway and allow for the degradation of enzymatic and non-enzymatic proteins along the spermatogenesis pathway. CRISPR, on the contrary, allows us to study genes involved with the different stages of spermatogenesis. The ideal targets are specific to the testes and have no paralogs.^138^ Table 3 shows a subset of promising targets for these technologies that have been separated into the stage of spermatogenesis where they function.^138^

**Table 3.** Selection of most promising non-hormonal target genes.

Stage of spermatogenesis	Target gene	Non-gonadal paralogs	Paralog similarity
Spermatogonia	*ASZ1*	Yes	<40%
	*MOV10L1*	Yes	<40%
	*TEX101*	Yes	<40%
	*SCML2*	Ubiquitous	60%
Spermatocyte	*MEIOB*	No	–
	*MEIOC*	No	–
	*PIH1D3*	No	–
	*SPATA22*	No	–
	*SYCE3*	No	–
	*TERB1*	No	–
	*TOPAZ1*	No	–
	*HORMAD2*	Yes	40–50%
	*HORMAD1*	Yes	40–50%
	*BTBD18*	Yes	<40%
	*CCDC63*	Yes	<40%
	*CCDC155*	Yes	<40%
	*HFM1*	Yes	<40%
	*MCMDC2*	Yes	<40%
	*TDRD5*	Yes	<40%
	*BOLL*	Yes	48–60%
	*FBXO43*	Yes	48–60%
	*INSL6*	Yes	48–60%
	*SOX30*	Yes	48–60%
	*SPDYA*	Yes	48–60%
	*LY6K*	Ubiquitous	28%
Round and elongated spermatids	*CCDC62*	No	–
	*SPACA1*	No	–
	*TCTE1*	No	–
	*TDRD12*	Yes	<40%
	*CCDC42*	Yes	<40%
	*PRSS37*	Yes	<40%
	*LRGUK*	Yes	<40%
	*CALR3*	Yes	51–55%
	*RIMBP3*	Yes	76%
	*RIMBP3B*	Yes	76%
	*RIMBP3C*	Yes	76%
Spermatozoa	*CATSPERD*	Yes	<50%
	*KCNU1*	Yes	<50%
	*PMIS2*	Yes	<50%
	*SUN5*	Yes	<50%
	*RSPH6A*	Yes	52–63%

## Reversible vasal occlusive products

### Implantable sperm valve

The Bimek SLV is an implantable device that occludes the vas with a reversible valve mechanism.^140^ The device is implanted in the outpatient setting, and the patient is able to operate the device with a palpable switch. It was first implanted in humans in 2009 and is currently undergoing further human clinical trials. This device theoretically allows patients to switch their fertility on and off but would require a wash out period each time the device is closed, similar to the waiting period required after vasectomy.

### Polymer injectables

Another area of active research is reversible vasal occlusion with polymer injectables. RISUG^®^ (reversible inhibition of sperm under guidance) is an ultrasound guided, sterile styrene maleic anhydride polymer injected into the vas. RISUG is approved in India for permanent sterilization and has been shown to be reversible in animal models.^141^ It is currently undergoing Phase III clinical trials. Vasalgel and Echo-V are two other polymer injectables currently in development.^142^ These are promising future products for reversible, procedural male sterilization.

## Conclusion

Reliable family planning is an essential element of modern society. For much of human history, the burden of unintended pregnancies have fallen disproportionately on the mother. Recent concerns regarding access to legal abortion has sparked a renewed interest for reliable male factor contraceptives beyond surgical sterilization and condoms. Modern efforts to develop a male birth control agent date back to the 1930s, and initially focused on altering the hypothalamic-pituitary-testes axis. Hormonal contraceptives face multiple barriers including systemic side effects, challenges with dosing regimens, route of delivery, and the public stigma of anabolic steroid abuse. Despite these challenges, some of the most widely studied and accepted potential male contraceptives are novel hormonal agents, such as DMAU and nestorone/testosterone gels. The recognition of retinoic acid receptors as potential reversible male contraceptives in the 1950s opened the door to exploring various non-hormonal agents. These novel non-hormonal contraceptives can target spermatogenesis at any stage of development, but common targets include Sertoli cell–germ cell interactions, sperm ion channels, testicular epigenetics, and an assortment of small molecule inhibitors. Several non-hormonal agents have entered human trials. The identification of reproductive tract–specific genes associated with male infertility, coupled with advances in PROTACs and CRISPR, may lead to more targeted drug development in the future. Despite multiple promising contenders over the last half century, no novel birth control agents have garnered regulatory approval in the United States or abroad. Future research is needed to achieve novel forms of male birth control.

## Declarations

**Ethics approval and consent to participate:** Not applicable.

**Consent for publication:** Not applicable.

**Availability of data and materials:** Not applicable.
